# Genetic association study of *TERT* gene variants with chronic kidney disease susceptibility in the Chinese population

**DOI:** 10.1080/0886022X.2023.2300725

**Published:** 2024-01-10

**Authors:** Yan Su, Yuan Feng, Xinran Lin, Chunyang Ma, Jiali Wei

**Affiliations:** a Department of Nephrology, Hainan General Hospital (Hainan Affiliated Hospital of Hainan Medical University), Haikou, PR China; b Department of Immunology, the Affiliated Children’s Hospital of Xi’an Jiaotong University, Xi’an, PR China; c Department of Nephrology, the First Affiliated Hospital of Hainan Medical College, Haikou, PR China; d Department of Neurosurgery, the First Affiliated Hospital of Hainan Medical College, Haikou, PR China

**Keywords:** Chronic kidney disease, genetic association study, TERT, gene variants

## Abstract

The incidence and mortality of chronic kidney disease (CKD) are increasing globally. Studies have demonstrated the significance of genetic risk factors in the progression of CKD. Telomerase reverse transcriptase (*TERT*) may be implicated in the development of CKD. This study aimed to investigate the correlation between *TERT* gene variants and susceptibility to CKD in the Chinese population. A total of 507 patients with CKD and 510 healthy controls were recruited for this case-control study. Four candidate loci were identified using the MassARRAY platform. Logistic regression analysis was employed to assess the association between *TERT* gene variants and the risk of CKD. The false positive reporting probability (FPRP) method was utilized to evaluate the validity of statistically significant associations. The multifactorial dimensionality reduction (MDR) method was used to evaluate the interaction between SNPs and the risk of CKD. Furthermore, discrepancies in the clinical features of subjects with diverse genotypes were evaluated using one-way analysis of variance (ANOVA). Our findings revealed a correlation between rs2735940 and rs4635969 and an increased risk of CKD. Stratification analysis indicated that rs4635969 was related to an increased risk of CKD in different subgroups (age ≤ 50 years and male). MDR analysis indicated that the two-site model (rs2735940 and rs4635969) was the best prediction model. Furthermore, the rs2735940 GG genotype was found to be linked to an increased level of microalbuminuria (MAU) in patients with CKD. Our study is the first to reveal a connection between *TERT* gene variants and susceptibility to CKD, providing new insights into the field of nephrology.

## Introduction

The kidney is a prominent excretory and endocrine organ that plays a vital role in maintaining body homeostasis by regulating fluid and electrolyte balance, blood pressure, and the composition and volume of blood [1]. Chronic kidney disease (CKD) is a long condition characterized by the gradual of kidney function over time. CKD is defined as the presence of an abnormality in kidney structure or function persisting for more than 3 months [2]. It is typically a progressive disease that can lead to kidney failure if left untreated. CKD affects about 10% of adults in many countries and is the common result of the continuous progression of various types of nephropathy [3]. CKD is expected to become the fifth leading cause of death by 2040 [4], which will impose a significant health and economic burden on the world. The study has shown that individuals with impaired renal function have an increased risk of progressing to ESRD [5], as well as an increased risk of morbidity and mortality from cardiovascular disease [6]. Simultaneously, hyperglycemia, hyperlipidemia, and hypertension are considered major risk factors that contribute to the progression of CKD [7]. Furthermore, studies on familial aggregation have shown that ESRD and early CKD tend to occur within families [8]. Studies have found the importance of other factors, including genetic risk factors, in the progression of nephropathy [9]. Therefore, studies based on susceptibility loci to unravel the genetic basis of nephropathy may lead to the discovery of new disease mechanisms.

Telomerase reverse transcriptase (TERT) serves as the catalytic subunit of telomerase. Its structure is highly conserved and plays a vital role in maintaining telomere and chromosome stability, as well as preventing malignant tumors [10,11]. It has been reported that abnormal telomerase activity is associated with the occurrence and development of cancer, and telomere length is negatively correlated with the incidence and mortality of cancer [12]. Some studies have found that carriers of short leukocyte telomere length exhibit a significant reduction in the GFR and an increase in the urinary albumin-to-creatinine ratio (ACR) [13,14]. *TERT* also plays a crucial role in the development and progression of kidney diseases. Several studies have provided evidence supporting the significance of *TERT* in renal pathologies. For example, a study Long et al. demonstrated that the upregulation of *TERT* promotes the growth of renal cell carcinoma (RCC) [15]. In addition, the *TERT* locus genotypes of rs2736100-CC/CA and rs2736098-AA have been associated with shorter survival in patients with RCC, as reported by Ma et al. [16]. Furthermore, the study by Casuscelli et al. focused on *TERT* promoter region mutations in RCC, and their findings suggest that *TERT* promoter mutations may serve as biomarkers for predicting the prognosis of RCC patients [17]. Moreover, there is a published literature report on epigenome-wide association study (EGWAS) for IgA nephropathy, and they found that three methylation CpGs corresponding to *TERT* were significantly associated with IgA nephropathy [18]. These findings indicate that *TERT* may play an important role in the pathogenesis of CKD. Single nucleotide polymorphisms (SNPs), the most common type of human heritable variants, are likely to be biomarkers for human disease, including nephropathy [19]. Previous study has showed that rs2736100 of *TERT* was associated with the risk of glomerulonephritis (GN), CDK, and ESRD [20]. However, the association between *TERT* gene variants and CKD has not been determined.

Therefore, this research aimed to investigate the association of *TERT* gene variants with CKD susceptibility in the Chinese population. Our study will help to elucidate the mechanism of *TERT* gene variants in the occurrence and progression of CKD, and provides new targets and strategies for the early diagnosis, prevention, and individualized treatment for CKD.

## Materials and methods

### Study subjects

The sample size was calculated using G* Power version 3.1.9.7 (Kiel, Germany) software prior to the study. Initially, the statistical method (*t*-test) was chosen, followed by the selection of classification (difference between two independent means (two groups)). The parameters were then set as follows: tail = 2, effective size = 0.2, *α* = 0.05, power = 0.885, and allocation ratio = 1. Finally, the sample size for both the case group and the control group was calculated to be 501 cases each. In this study, a total of 1017 unrelated Chinese Han participants, including 507 CKD patients and 510 healthy subjects, were randomly selected from the First Affiliated Hospital of Xi’an Jiaotong University between April 2018 and December 2021. This article fully complies with the principles of the Declaration of Helsinki and has been approved by the Ethics Committee of the First Affiliated Hospital of Hainan Medical College. Informed consent was obtained from all participants prior to the start of the study. All patients were initially diagnosed with CKD and confirmed by two experienced nephrologists through the standard clinical diagnosis, laboratory tests, imaging, or histopathological evaluation. The inclusion criteria for the patients are as follows: (1) age ≥ 18 years old; (2) patients were confirmed CKD by the diagnostic criteria for CKD; (3) patients were Chinese Han population. The criteria for CKD diagnosis are as follows: 1) a glomerular filtration rate (GFR) of less than 60 mL/min/1.73 m^2^, albuminuria of at least 30 mg per 24 h, or urine ACR ≥ 30 mg/g), or 2) markers of kidney damage (e.g., hematuria or structural abnormalities, such as polycystic or dysplastic kidneys) persisting for more than 3 months, or 3) renal tubular disorders [21]. Staging of GFR is classified as G1 (GFR ≥ 90 mL/min/1.73 m^2^), G2 (GFR 60–89 mL/min/1.73 m^2^), G3 (30–59 mL/min/1.73 m^2^), G4 (15–29 mL/min/1.73 m^2^), and G5 (<15 mL/min/1.73 m^2^). The exclusion criteria for the patients were as follows: (1) patients with genetic diseases; (2) patients with any medical or family history of kidney damage or CKD (3) patients who had previously received renal replacement therapy patients with established nephropathy; (4) patients with other major diseases or comorbidities, such as malignant tumors or heart diseases (including arterial hypertension, left ventricular hypertrophy/diastolic dysfunction, and coronary artery disease); and (5) patients for whom complete clinical data were not available. The inclusion criteria for the control group were as follows: (1) healthy individuals with normal renal function who underwent a physical examination at the same hospital as the cases during the corresponding period; (2) age ≥ 18 years old; and (3) gender and other characteristics matched to the case group. The exclusion criteria for the control group were as follows: (1) a history of nephropathy or any other serious illness; (2) patients for whom complete clinical data were not available; and (3) the presence of a familial relationship with the case group. Moreover, the basic features of the subjects, such as age, gender, and CKD stage, were assessed through questionnaires or by professional physicians.

### SNP selection and genotyping

The SNPs of *TERT* were chosen from the 1000 Genomes Project based on the following criteria: minor allele frequency (MAF) > 0.05, Hardy–Weinberg Equilibrium (HWE)-*p* > 0.05, and Tagger *r*^2^ < 0.8. Rs2736100 C/A, rs2853677 G/A, rs2735940 A/G, and rs4635969 A/G were selected for follow-up research. Genomic DNA was extracted using a DNA extraction kit following the manufacturer’s instructions (GoldMag Co. Ltd., Xi’an, China). The extracted DNA was then stored at −80 °C in an ultra-low temperature freezer. The primers were designed using MassARRAY Assay Design software. SNPs were genotyped using the MassARRAY system (Agena, San Diego, CA). SNP genotypes were identified using iPLEX chemistry. MALDI-TOF was used to obtain mass peak profiles for various reactions, and genotyping was subsequently successfully completed.

### Statistical analysis

Baseline features of the subjects were analyzed using SPSS version 21.0 (SPSS, Chicago, IL). Among them, age and gender were analyzed by *t*-test and *χ*^2^ test, respectively. The genotype frequencies of all SNPs were obtained using a Chi-square test to determine if they satisfied HWE. Odds ratios (ORs) and 95% confidence intervals (95% CIs) were calculated using logistic regression analysis, considering different genetic models, to evaluate the association between all SNPs and the risk of CKD. Multiple genetic models were evaluated using PLINK version 1.9 (Massachusetts, USA), with wild-type alleles serving as the reference. Moreover, the results were analyzed using the false positive reporting probability (FPRP) test to determine if they warranted attention. The multifactorial dimensionality reduction (MDR) method was used to assess the interaction of four SNPs with the risk of CKD. Discrepancies in clinical features among subjects with diverse genotypes were compared using one-way analysis of variance (ANOVA).

## Results

### The basic characteristics of study participants

Baseline features of the control and case groups are presented in Table 1. There were 1017 unrelated subjects in our research, including 507 CKD patients with a mean age of 49.40 ± 16.76 years and 510 healthy individuals with a mean age of 49.40 ± 16.09 years. The proportion of females (38%) was equal in both groups. The mean age (*p* = 0.999) and gender distribution (*p* = 0.941) of the two groups were exactly matched. Moreover, the levels of microalbuminuria (MAU), uric acid (UA), creatinine (Cr), and urea were significantly higher in patients with CKD compared to the control group.

**Table 1.** Characteristics of patients with chronic kidney disease and healthy individuals.

Characteristics	Cases, *n* = 507	Control, *n* = 510	*p*
Age (years, mean ± SD)	49.40 ± 16.76	49.40 ± 16.09	0.999
> 50	265 (52%)	267 (52%)	
≤ 50	242 (48%)	243 (48%)	
Gender			0.941
Male	312 (62%)	315 (62%)	
Female	195 (38%)	195 (38%)	
MAU (mg/L)	616.22 ± 845.45	29.81 ± 30.09	**< 0.001**
UA (umol/L)	402.57 ± 135.45	331.37 ± 90.07	**< 0.001**
Creatinine (umol/L)	409.39 ± 443.26	66.18 ± 13.83	**< 0.001**
Urea (mmol/L)	13.88 ± 19.06	4.92 ± 1.26	**< 0.001**
CKD stage			
CKD1	154	–	–
CKD2	100	–	–
CKD3	45	–	–
CKD4	22	–	–
CKD5	164	–	–
Missing	22	–	–

MAU: microalbuminuria; UA: uric acid; CKD: chronic kidney disease

*p* < 0.05 and bold text indicates statistical significance.

### Genotyping information

**Figure 1.**
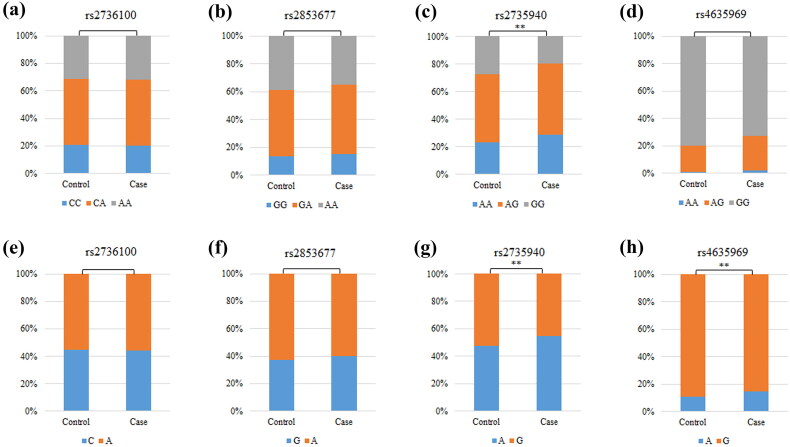
Comparisons of the genotype frequency and allele frequency of SNPs in the *TERT* gene between the CKD case and healthy control groups. (a–d) Comparisons of the genotype frequencies of rs2736100, rs2853677, rs2735940, and rs4635969. (e–h) Comparisons of the allele frequencies of rs2736100, rs2853677, rs2735940, and rs4635969. ‘*’ indicates statistical significance at the 0.05 level.

Detailed genotyping information for all SNPs is provided in Table 2. The genotypes of four SNPs were in accordance with HWE (*p* > 0.05), and MAF was greater than 5% in the study population. Four SNPs were predicted by HaploReg to be regulated by Motifs changed, NHGRI/EBI GWAS hit, Promoter histone marks, Enhancer histone marks, and GRASP QTL hits. Moreover, we discovered discrepancies in the genotype frequency and allele frequency of rs2735940 (*p* = 0.008 and *p* = 0.002, respectively), as well as the allele frequency of rs4635969 (*p* = 0.005), between CKD patients and healthy controls (Figure 1).

**Table 2.** The basic information and HWE about the selected SNPs.

Gene	SNP ID	Chrs	Position	Function	Alleles (A/B)	MAF		HWE (*p* value)	Haploreg 4.2
						Cases	Control		
*TERT*	rs2736100	5	1286401	intronic	C/A	0.442	0.446	0.531	Motifs changed, NHGRI/EBI GWAS hits, GRASP QTL hits
*TERT*	rs2853677	5	1287079	intronic	G/A	0.402	0.374	0.850	NHGRI/EBI GWAS hits
*TERT*	rs2735940	5	1296371	–	A/G	0.546	0.478	1.000	Promoter histone marks, Enhancer histone marks
*TERT*	rs4635969	5	1308437	–	A/G	0.147	0.106	0.638	Enhancer histone marks, Motifs changed, NHGRI/EBI GWAS hits

HWE: Hardy–Weinberg equilibrium; SNP: single nucleotide polymorphisms; Chrs: chromosome number; Alleles (A/B): minor/major allele; MAF: minor allele frequency

*p* > 0.05 indicates that the genotypes were in Hardy–Weinberg Equilibrium.

### Correlation between *TERT* gene variants and susceptibility to CKD

**Figure 2.**
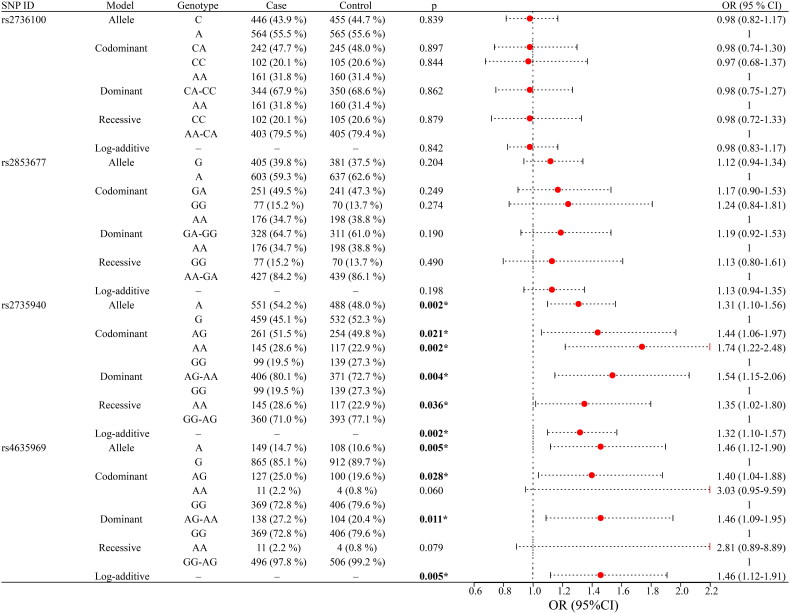
Analysis of the association between susceptibility to CKD and SNPs. SNPs: single nucleotide polymorphisms; OR: odds ratio; CI: confidence interval; *p* values represent adjustment for age and gender; *p* < 0.05, bold text and ‘*’ indicate statistical significance.

The correlation between four SNPs and susceptibility to CKD is presented in Figure 2. The results indicated that rs2735940 and rs4635969 were significantly associated with susceptibility to CKD. Specifically, rs2735940 was significantly associated with an increased risk of CKD under the following models: allele (A *vs.* G: OR = 1.31, 95% CI 1.10–1.56, *p* = 0.002), heterozygote (AG *vs.* GG: OR = 1.44, 95% CI 1.06–1.97, *p* = 0.021), homozygote (AA *vs.* GG: OR = 1.74, 95% CI 1.22–2.48, *p* = 0.002), dominant (AG-AA *vs.* GG: OR = 1.54, 95% CI 1.15–2.06, *p* = 0.004), recessive (AA *vs.* GG-AG: OR = 1.35, 95% CI 1.02–1.80, *p* = 0.036), and log-additive (OR = 1.32, 95% CI 1.10–1.57, *p* = 0.002) models. Similarly, rs4635969 was notably linked to an increased risk of CKD under the allele (A *vs.* G: OR = 1.46, 95% CI 1.12–1.90, *p* = 0.005), codominant (AG *vs*. GG: OR = 1.40, 95% CI 1.04–1.88, *p* = 0.028), dominant (AG-AA *vs*. GG: OR = 1.46, 95% CI 1.09–1.95, *p* = 0.011), and log-additive (OR = 1.46, 95% CI 1.12–1.91, *p* = 0.005) models.

### Correlation between *TERT* gene variants and the risk of CKD in different stratifications

The correlation between four SNPs and CKD risk stratified by age and gender is listed in Table 3. We discovered that rs2735940 is linked to an increased risk of CKD in both subjects aged ≤ 50 years (allele: OR = 1.29, 95% CI 1.00–1.67, *p* = 0.046; codominant: OR = 1.68, 95% CI 1.00–2.81, *p* = 0.048; and log-additive: OR = 1.30, 95% CI 1.00–1.68, *p* = 0.048) and subjects aged > 50 years (allele: OR = 1.32, 95% CI 1.04–1.69, *p* = 0.023; codominant: OR = 1.82, 95% CI 1.11–2.99, *p* = 0.018; dominant: OR = 1.63, 95% CI 1.07–2.47, *p* = 0.023; and log-additive: OR = 1.34, 95% CI 1.05–1.71, *p* = 0.020). And it was also linked to an increased risk of CKD in both male participants (allele: OR = 1.27, 95% CI 1.02–1.59, *p* = 0.035; heterozygote: OR = 1.57, 95% CI 1.05–2.33, *p* = 0.027; homozygote: OR = 1.64, 95% CI 1.05–2.56, *p* = 0.031; dominant: OR = 1.59, 95% CI 1.09–2.32, *p* = 0.015; and log-additive: OR = 1.27, 95% CI 1.02–1.59, *p* = 0.036) and female participants (allele: OR = 1.38, 95% CI 1.04–1.82, *p* = 0.026; codominant: OR = 1.98, 95% CI 1.10–3.56, *p* = 0.023; recessive: OR = 1.67, 95% CI 1.04–2.68, *p* = 0.033; and log-additive: OR = 1.41, 95% CI 1.05–1.89, *p* = 0.023). Furthermore, rs4635969 was tied to an increased risk of CKD in individuals aged ≤ 50 years under a variety of genetic models (allele: OR = 1.62, 95% CI 1.09–2.40, *p* = 0.016; dominant: OR = 1.59, 95% CI 1.03–2.46, *p* = 0.036; and log-additive: OR = 1.59, 95% CI 1.08–2.36, *p* = 0.020). And it was also a risk factor in male participants (allele: OR = 1.61, 95% CI 1.14–2.28, *p* = 0.006; codominant: OR = 1.55, 95% CI 1.05–2.27, *p* = 0.027; dominant: OR = 1.63, 95% CI 1.12–2.37, *p* = 0.012; and log-additive: OR = 1.62, 95% CI 1.15–2.30, *p* = 0.006). When stratified by CKD stage, it was observed that there was no significant correlation between *TERT* gene variants and the susceptibility to CKD (Table S1).

**Table 3.** The SNPs associated with susceptibility to chronic kidney disease in the subgroup tests (age and gender).

SNP ID	Model	Genotype	Age				Gender			
			**≤** 50 (*N* = 485)	*p*	> 50 (*N* = 532)	*p*	Male (*N* = 627)	*p*	Female (*N* = 390)	*p*
			OR (95% CI)		OR (95% CI)		OR (95% CI)		OR (95% CI)	
rs2736100	Allele	C	0.91 (0.71–1.17)	0.468	1.05 (0.83–1.34)	0.681	0.96 (0.77–1.20)	0.718	1.02 (0.77–1.36)	0.893
		A	1		1		1		1	
	Codominant	CA	0.90 (0.60–1.36)	0.614	1.06 (0.72–1.57)	0.754	1.01 (0.71–1.46)	0.942	0.94 (0.60–1.46)	0.770
		CC	0.83 (0.50–1.38)	0.484	1.11 (0.68–1.81)	0.673	0.92 (0.59–1.42)	0.695	1.08 (0.60–1.94)	0.805
		AA	1		1		1		1	
	Dominant	CA-CC	0.88 (0.60–1.29)	0.513	1.08 (0.75–1.56)	0.690	0.98 (0.70–1.38)	0.915	0.97 (0.63–1.48)	0.890
		AA	1		1		1		1	
	Recessive	CC	0.89 (0.57–1.38)	0.602	1.07 (0.70–1.64)	0.757	0.91 (0.62–1.33)	0.621	1.12 (0.67–1.89)	0.667
		AA-CA	1		1		1		1	
	Log-additive	–	0.91 (0.71–1.17)	0.471	1.06 (0.83–1.34)	0.663	0.96 (0.77–1.20)	0.725	1.02 (0.77–1.36)	0.886
rs2853677	Allele	G	1.09 (0.84–1.41)	0.518	1.15 (0.90–1.48)	0.254	1.15 (0.91–1.44)	0.244	1.09 (0.81–1.46)	0.566
		A	1		1		1		1	
	Codominant	GA	1.11 (0.75–1.63)	0.605	1.24 (0.85–1.80)	0.267	1.29 (0.91–1.82)	0.152	1.01 (0.66–1.56)	0.962
		GG	1.19 (0.67–2.11)	0.548	1.30 (0.77–2.19)	0.320	1.23 (0.76–1.98)	0.394	1.28 (0.67–2.44)	0.458
		AA	1		1		1		1	
	Dominant	GA-GG	1.13 (0.78–1.63)	0.532	1.25 (0.88–1.79)	0.215	1.27 (0.92–1.76)	0.147	1.06 (0.70–1.60)	0.780
		AA	1		1		1		1	
	Recessive	GG	1.12 (0.66–1.90)	0.663	1.15 (0.72–1.85)	0.556	1.07 (0.69–1.65)	0.770	1.27 (0.70–2.31)	0.433
		AA-GA	1		1		1		1	
	Log-additive	–	1.10 (0.84–1.43)	0.502	1.16 (0.91–1.49)	0.240	1.15 (0.91–1.44)	0.243	1.10 (0.81–1.48)	0.549
rs2735940	Allele	A	1.29 (1.00–1.67)	0.046*	1.32 (1.04–1.69)	0.023*	1.27 (1.02–1.59)	0.035*	1.38 (1.04–1.82)	0.026*
		G	1		1		1		1	
	Codominant	AG	1.37 (0.88–2.12)	0.164	1.53 (0.99–2.38)	0.058	1.57 (1.05–2.33)	0.027*	1.27 (0.77–2.09)	0.343
		AA	1.68 (1.00–2.81)	0.048*	1.82 (1.11–2.99)	0.018*	1.64 (1.05–2.56)	0.031*	1.98 (1.10–3.56)	0.023*
		GG	1		1		1		1	
	Dominant	AG-AA	1.46 (0.96–2.21)	0.075	1.63 (1.07–2.47)	0.023*	1.59 (1.09–2.32)	0.015*	1.46 (0.91–2.34)	0.120
		GG	1		1		1		1	
	Recessive	AA	1.36 (0.90–.08)	0.149	1.35 (0.92–1.98)	0.126	1.20 (0.85–1.72)	0.304	1.67 (1.04–2.68)	0.033*
		GG-AG	1		1		1		1	
	Log-additive	–	1.30 (1.00–1.68)	0.048*	1.34 (1.05–1.71)	0.020*	1.27 (1.02–1.59)	0.036*	1.41 (1.05–1.89)	0.023*
rs4635969	Allele	A	1.62 (1.09–2.40)	0.016*	1.33 (0.93–1.91)	0.113	1.61 (1.14–2.28)	0.006*	1.25 (0.83–1.89)	0.292
		G	1		1		1		1	
	Codominant	AG	1.49 (0.95–2.33)	0.084	1.34 (0.90–2.00)	0.151	1.55 (1.05–2.27)	0.027*	1.20 (0.75–1.93)	0.439
		AA	3.86 (0.79–18.82)	0.095	2.21 (0.40–12.24)	0.363	3.94 (0.81–19.17)	0.089	2.12 (0.38–11.77)	0.391
		GG	1		1		1		1	
	Dominant	AG-AA	1.59 (1.03–2.46)	0.036*	1.37 (0.92–2.03)	0.117	1.63 (1.12–2.37)	0.012*	1.24 (0.79–1.97)	0.351
		GG	1		1		1		1	
	Recessive	AA	3.56 (0.73–17.34)	0.116	2.06 (0.37–11.35)	0.408	3.60 (0.74–17.45)	0.112	2.03 (0.37–11.22)	0.419
		GG-AG	1		1		1		1	
	Log-additive	–	1.59 (1.08–2.36)	0.020*	1.36 (0.94–1.97)	0.099	1.62 (1.15–2.30)	0.006*	1.26 (0.83–1.91)	0.288

SNP: single nucleotide polymorphisms; OR: odds ratio; CI: confidence interval

*p* < 0.05, bold text and ‘*’ indicate statistical significance.

### FPRP results

The results of FPRP analysis can be found in Table S2. The FPRP values were all less than 0.2 at a prior probability level of 0.25, indicating that the positive results were noteworthy. The overall analysis findings were entirely noteworthy and the stratification analysis findings were almost noteworthy at a prior probability level of 0.25 and an FPRP value of 0.2.

### MDR results

**Figure 3.**
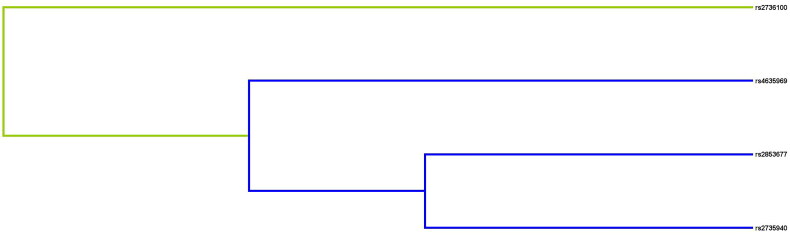
Dendrogram of SNP-SNP interactions. The colors in the tree diagram represent either synergy or redundancy.

The results of MDR analysis were carried out to evaluate SNP-SNP interactions (Figure 3). The blue lines in the dendrogram indicate a strong redundant effect of rs4635969, rs2853677, and rs2735940 on the risk of CKD. Details of SNP-SNP interactions are listed in Table 4. We discovered that the best prediction model was the two-site model: rs2735940 and rs4635969 (the largest CVC: 10/10, with the largest testing balanced accuracy of 0.559 (*p* < 0.0001).

**Table 4.** SNP–SNP interaction models analyzed by the MDR method.

Model	Training Bal. Acc	Testing Bal. Acc	*p* Value	CVC
rs2735940	0.540	0.519	** 0.0038**	8/10
rs2735940, rs4635969	0.564	0.559	***p* < 0.0001**	10/10
rs2736100, rs2735940, rs4635969	0.570	0.516	***p* < 0.0001**	9/10
rs2736100, rs2853677, rs2735940, rs4635969	0.581	0.524	***p* < 0.0001**	10/10

MDR: multifactor dimensionality reduction; Bal. Acc.: balanced accuracy; CVC: cross-validation consistency.

*p* values were calculated using *χ*^2^ tests; *p* < 0.05 and bold text indicate statistical significance.

### The correlation of *TERT* gene variants with clinical indicators

The correlation between *TERT* gene variants and clinical indicators of CKD patients was conducted, as presented in Table 5. Compared to the GA genotype, individuals with the rs2735940 AA/GG genotype were found to have a higher level of MAU (*p* = 0.023). There was no relevance between the remaining three variants and the levels of FPG, MAU, LDL, UA, Cr, and urea (*p* > 0.05).

**Table 5.** Clinical characteristics of patients (*N* = 507) based on the genotypes of selected SNPs.

	MAU	UA	Creatinine	Urea
rs2736100				
AA	684.24 ± 1044.44	412.99 ± 136.42	383.46 ± 388.40	13.62 ± 9.00
CA	578.15 ± 680.48	399.34 ± 131.05	441.32 ± 509.88	12.94 ± 8.25
CC	579.42 ± 779.71	395.58 ± 144.64	370.46 ± 342.56	16.41 ± 39.23
*p*	0.575	0.522	0.290	0.320
rs2853677				
AA	655.17 ± 930.86	415.64 ± 134.65	387.62 ± 395.33	13.11 ± 8.56
GA	604.39 ± 806.75	395.87 ± 138.49	431.73 ± 498.38	13.01 ± 8.39
GG	566.20 ± 742.23	398.66 ± 124.20	380.01 ± 343.46	18.76 ± 45.05
*p*	0.798	0.332	0.512	0.064
rs2735940				
AA	577.18 ± 878.21	397.08 ± 128.38	397.47 ± 553.65	15.83 ± 33.37
GA	532.39 ± 638.66	405.81 ± 135.13	419.43 ± 393.34	12.88 ± 8.10
GG	870.10 ± 1148.92	405.07 ± 146.16	398.70 ± 390.69	13.85 ± 9.12
*p*	0.023*	0.822	0.868	0.348
rs4635969				
AA	668.19 ± 370.72	360.27 ± 117.26	365.64 ± 411.09	9.65 ± 6.22
GA	603.59 ± 837.23	394.65 ± 138.18	395.23 ± 361.02	13.67 ± 8.77
GG	618.97 ± 860.88	406.74 ± 134.99	415.80 ± 470.80	14.09 ± 21.82
*p*	0.977	0.401	0.858	0.742

MAU: microalbuminuria; UA: uric acid

*p* < 0.05, bold text and ‘*’ represent statistical significance.

## Discussion

In this study, we examined the association between four SNPs in the *TERT* gene and the risk of CKD in a Chinese population. The results showed that rs2735940 and rs4635969 were associated with an increased risk of CKD. This study is the first and largest case-control study to date on four SNPs of *TERT* and their association with the risk of developing CKD.

Increasing evidence has shown that *TERT* gene variants are associated with the risk of developing, surviving, and prognosing various cancers [22–24]. However, there are relatively few studies on the effect of *TERT* gene variants on the risk of CKD. Although this association signal was reported in a recent association study, it included only one SNP of *TERT*, rs2736100 [20]. Interestingly, in this study, we did not identify this signal. The variation in the results of the aforementioned studies could be attributed to several factors, including the limited impact of this genetic variants on CKD, the relatively small sample size in each study, and the heterogeneity of the population [25]. The role of *TERT* variants in CKD is not yet well understood. Therefore, the aim of this study was to investigate the association between *TERT* gene polymorphisms and susceptibility to CKD. Our study demonstrated that rs2735940 and rs4635969 were associated with an increased risk of CKD. Our study is the first to reveal a connection between TERT polymorphisms and susceptibility to CKD, providing new insights into the field of nephrology.

There have been numerous studies conducted on rs2735940, with the majority of them concentrating on the correlation between rs2735940 and the susceptibility to different types of cancer. To the best of our knowledge, no studies have reported genetic association studies between the SNP rs2735940 and CKD. However, a study has found that the *TERT* rs2735940 affects early renal function after transplantation [26]. The study by Sheng et al. has shown that the *TERT* rs2735940 is a functional SNP both *in vitro* and *in vivo*, which may affect *TERT* mRNA expression by influencing transcriptional activity [27]. Thus, this polymorphism may lead to a reduced expression of *TERT*. Lower *TERT* levels could result in shorter telomeres and accelerated cellular aging, making the kidneys more vulnerable to damage and CKD. Thus, the potential influence of rs2735940 on the development of CKD may be attributed to impact on the expression of *TERT*. The results of our study indicate that individuals with the A, AG, and AA genotypes had a higher risk of developing CKD compared to those with other genotypes. Furthermore, our study discovered that individuals with the A and AG genotype of rs4635969 in *TERT* are at a heightened risk of developing CKD in comparison to individuals with other genotypes. These findings potentially have implications for early CKD diagnosis, personalized treatment approaches, and preventive measures. Analyzing an individual’s genotype can enable us to predict their likelihood of developing CKD and implement appropriate preventive measures or early treatments. However, it is important to note that these results are preliminary, and further research, including larger cohorts and functional studies, is needed to validate the clinical relevance and applicability of these SNPs in CKD diagnosis. Although there is currently no report on the biological function of rs4635969, we used Haploreg4.2 online software to predict its potential functions and found that rs4635969 could influence the regulation of enhancer histone marks and Motifs changed. Taken above, we speculated that *TERT* SNPs, especially rs2735940 and rs4635969 may affect gene expression and function, and then contribute to CKD development, which needs further study and confirmation. Moreover, the identification of genetic markers like rs2735940 and rs4635969 in understanding the underlying genetic predispositions to these conditions. This understanding can lead to better risk assessment, early detection, and potentially more targeted therapeutic strategies.

Age could contribute to the progression of CKD [28]. Meanwhile, gender has historically been regarded as an important factor affecting renal function and the progression of CKD [29]. Therefore, we stratified by age and gender to investigate the relationship between *TERT* variants and susceptibility to CKD. In our study, we found that rs4635969 is associated with an increased risk of CKD in individuals aged ≤ 50 years. The results of Kobayashi et al. have demonstrated that genetic variants are associated with renal function in an elderly Japanese population (mean age 73 years) [30], which is inconsistent with our findings. The evidence above suggests that genetic variants and susceptibility to CKD may be influenced by age. Our study found that rs4635969 was associated with an increased risk of CKD in males. Cumulative evidence from epidemiological and clinical studies has suggested that there may be gender-related differences in the prevalence, course, and severity of CKD [31]. Consistent with our findings, the study by Chung et al. found that genetic variants were associated with the progression of nephropathy in Taiwanese males [32]. A case-control study by Niu et al. showed that genetic variants were associated with an increased risk of nephropathy in males [33]. In contrast to our findings, Sun et al. have reported that variants in the telomerase RNA component (*TERC*) gene were associated with GN, CKD, and ESRD risk only in females [20]. A plausible explanation for this phenomenon is that estrogen and progesterone can activate the transcription of *TERT* and *TERC* genes [34,35], and it is speculated that female sex hormones play a significant role in this process. Therefore, there are gender differences in genetic variants and susceptibility to CKD.

Additionally, studies have shown that MAU is an indicator of kidney damage and a risk factor for the progression of nephropathy, mainly reflects glomerular damage and glomerular permeability to macromolecules [36]. The presence of MAU is indicative of early nephropathy, and both the absolute level and rate of change of MAU are associated with the development and progression of diabetic kidney disease [37]. Our study found that that individuals with the rs2735940 GG genotype was linked to an elevated level of MAU in patients with CKD. Therefore, rs2735940 may be a risk factor for CKD.

However, it is important to note that our study was limited to a Chinese population. The study has found that the *TERT* variant has a different genotype distribution in Swedish and Chinese populations [38]. The prevalence of primary GN, CKD, and ESRD in China is significantly higher than in European and American countries [39]. Therefore, different genotype distributions of *TERT* variants may lead to varying susceptibility to CKD. Further studies will be conducted to investigate the association between *TERT* variants and susceptibility to CKD in different populations, aiming to provide a more precise understanding of this possibility. Despite the above limitation, our study is the first to reveal a connection between *TERT* variants and susceptibility to CKD, providing new insights into the field of nephrology.

## Conclusion

In summary, this study demonstrated that rs2735940 and rs4635969 in the *TERT* gene variants were significantly associated with an increased susceptibility to CKD in the Chinese population. Our findings could potentially change nephrologists’ understanding of disease susceptibility and offer new strategies for potential risk assessment, prevention, and early intervention.

## Supplementary Material

Supplemental Material

## Data Availability

The original contributions or analyzed during this study are available from the article. Further inquiries can be made to the corresponding author.
