# Supplementary material for: Genetic association study of TERT gene variants with chronic kidney disease susceptibility in the Chinese population
Source: Ren Fail. 2024 Jan 10;46(1):2300725. doi: 10.1080/0886022X.2023.2300725 (PMC10783823; doi:10.1080/0886022X.2023.2300725)
Supplement: Supplemental Material [file IRNF_A_2300725_SM2718.pdf]

Table S1 The association between *TERT* variants and CKD susceptibility stratified by CKD stage

SNP ID	Model	Genotype	CKD 4-5 vs CKD 1-3	
			OR (95% CI)	*p*
rs2736100	Allele	C	1.09 (0.84-1.41)	0.548
		A	1	
	Codominant	CA	1.23 (0.86-1.77)	0.264
		CC	1.14 (0.67-1.96)	0.631
		AA	1	
	Dominant	CA-CC	1.12 (0.74-1.68)	0.588
		AA	1	
	Recessive	CC	1.07 (0.67-1.72)	0.771
		AA-CA	1	
	Log-additive	–	1.07 (0.82-1.40)	0.604
rs2853677	Allele	G	1.00 (0.77-1.30)	1.000
		A	1	
	Codominant	GA	0.93 (0.62-1.42)	0.758
		GG	0.96 (0.54-1.72)	0.894
		AA	1	
	Dominant	GA-GG	0.94 (0.63-1.40)	0.769
		AA	1	
	Recessive	GG	1.00 (0.60-1.69)	0.998
		AA-GA	1	
	Log-additive	–	0.97 (0.74-1.28)	0.837
rs2735940	Allele	A	1.19 (0.91-1.54)	0.207
		G	1	
	Codominant	AG	1.34 (0.86-2.11)	0.199
		AA	1.53 (0.88-2.66)	0.134
		GG	1	
	Dominant	AG-AA	1.39 (0.91-2.14)	0.128
		GG	1	
	Recessive	AA	1.26 (0.79-2.02)	0.331
		GG-AG	1	
	Log-additive	–	1.24 (0.95-1.64)	0.120
rs4635969	Allele	A	1.23 (0.86-1.77)	0.264
		G	1	
	Codominant	AG	1.22 (0.79-1.88)	0.379
		AA	1.67 (0.48-5.85)	0.420
		GG	1	
	Dominant	AG-AA	1.25 (0.82-1.91)	0.301
		GG	1	
	Recessive	AA	1.59 (0.46-5.54)	0.464
		GG-AG	1	
	Log-additive	–	1.24 (0.85-1.80)	0.258

SNP, Single nucleotide polymorphisms; OR, odds ratio; CI, Confidence interval.

Information Classification: General

Table S2 The FPRP and statistical power values of the positive results in this study.

SNP ID	Model	Genotype	OR (95 % CI)	*p*	Statistical Power[a] (%)	Prior probability		
						0.25	0.1	0.01
**Overall analysis**								
rs2735940	Allele	A	1.31 (1.10-1.56)	0.002	0.936	0.008 [b]	0.023 [b]	0.205
	Codominant	AG	1.44 (1.06-1.97)	0.021	0.601	0.101 [b]	0.253	0.788
		AA	1.74 (1.22-2.48)	0.002	0.206	0.031 [b]	0.087 [b]	0.513
	Dominant	AG-AA	1.54 (1.15-2.06)	0.004	0.430	0.025 [b]	0.071 [b]	0.455
	Recessive	AA	1.35 (1.02-1.80)	0.036	0.764	0.138 [b]	0.325	0.841
	Log-additive	–	1.32 (1.10-1.57)	0.002	0.933	0.021 [b]	0.060 [b]	0.414
rs4635969	Allele	A	1.46 (1.12-1.90)	0.005	0.580	0.025 [b]	0.070 [b]	0.454
	Codominant	AG	1.40 (1.04-1.88)	0.028	0.677	0.101 [b]	0.252	0.787
	Dominant	AG-AA	1.46 (1.09-1.95)	0.011	0.573	0.052 [b]	0.140 [b]	0.642
	Log-additive	–	1.46 (1.12-1.91)	0.005	0.578	0.029 [b]	0.082 [b]	0.497
**Age ≤ 50**								
rs2735940	Allele	A	1.29 (1.00-1.67)	0.046	0.874	0.154 [b]	0.354	0.858
	Codominant	AA	1.68 (1.00-2.81)	0.048	0.333	0.302	0.565	0.935
	Log-additive	–	1.30 (1.00-1.68)	0.048	0.863	0.135 [b]	0.319	0.837
rs4635969	Allele	A	1.62 (1.09-2.40)	0.016	0.351	0.121 [b]	0.293	0.820
	Dominant	AG-AA	1.59 (1.03-2.46)	0.036	0.397	0.220	0.458	0.903
	Log-additive	–	1.59 (1.08-2.36)	0.020	0.386	0.142 [b]	0.332	0.846
**Age > 50**								
rs2735940	Allele	A	1.32 (1.04-1.69)	0.023	0.845	0.089 [b]	0.228	0.764
	Codominant	AA	1.82 (1.11-2.99)	0.018	0.223	0.196 [b]	0.422	0.889
	Dominant	AG-AA	1.63 (1.07-2.47)	0.023	0.348	0.155 [b]	0.355	0.858
	Log-additive	–	1.34 (1.05-1.71)	0.020	0.818	0.064 [b]	0.170 [b]	0.693

Information Classification: General

**Male**								
rs2735940	Allele	A	1.27 (1.02-1.59)	0.035	0.927	0.107 [b]	0.265	0.798
	Codominant	AG	1.57 (1.05-2.33)	0.027	0.410	0.155 [b]	0.355	0.858
		AA	1.64 (1.05-2.56)	0.031	0.347	0.203	0.433	0.894
	Dominant	AG-AA	1.59 (1.09-2.32)	0.015	0.381	0.113 [b]	0.276	0.807
	Log-additive	–	1.27 (1.02-1.59)	0.036	0.927	0.107 [b]	0.265	0.798
rs4635969	Allele	A	1.61 (1.14-2.28)	0.006	0.345	0.060 [b]	0.160 [b]	0.677
	Codominant	AG	1.55 (1.05-2.27)	0.027	0.433	0.144 [b]	0.336	0.848
	Dominant	AG-AA	1.63 (1.12-2.37)	0.012	0.332	0.087 [b]	0.222	0.758
	Log-additive	–	1.62 (1.15-2.30)	0.006	0.333	0.059 [b]	0.158 [b]	0.674
**Female**								
rs2735940	Allele	A	1.38 (1.04-1.82)	0.026	0.723	0.086 [b]	0.219	0.755
	Codominant	AA	1.98 (1.10-3.56)	0.023	0.177	0.276	0.534	0.926
	Recessive	AA	1.67 (1.04-2.68)	0.033	0.328	0.235	0.479	0.910
	Log-additive	–	1.41 (1.05-1.89)	0.023	0.661	0.089 [b]	0.227	0.763

FPRP, false-positive report probability; SNP, single nucleotide polymorphism; OR, Odds ratio; CI, Confidence interval.

[a] Statistical power[a] was calculated using the number of the OR and *p* values in this table.

[b] The level of false-positive report probability threshold was set at 0.2, and noteworthy findings are presented.

Information Classification: General
